# Transcriptome changes of fission yeast cells exposed to fumonisin B1 or co-cultured with *Fusarium verticillioides*

**DOI:** 10.1007/s00253-025-13601-3

**Published:** 2025-10-01

**Authors:** László Attila Papp, Lajos Acs-Szabo, Szilvia Kovács, Cintia Adácsi, Gyula Batta, Tünde Pusztahelyi, István Pócsi, Ida Miklós

**Affiliations:** 1 Department of Genetics and Applied Microbiology, Faculty of Science and Technology, Institute of Biotechnology, University of Debrecen, Egyetem Tér 1, 4032 Debrecen, Hungary; 2 Department of Botany, Faculty of Science and Technology, Institute of Biology and Ecology, University of Debrecen, Egyetem Tér 1, 4032 Debrecen, Hungary; 3 Present Address: Central Laboratory of Agricultural and Food Products, Faculty of Agricultural and Food Sciences and Environmental Management, University of Debrecen, Böszörményi Street 138, 4032 Debrecen, Hungary; 4 Department of Molecular Biotechnology and Microbiology, Faculty of Science and Technology, Institute of Biotechnology, University of Debrecen, Egyetem Tér 1, 4032 Debrecen, Hungary; 5 HUN-REN-UD Fungal Stress Biology Research Group, Egyetem Tér 1, 4032 Debrecen, Hungary

**Keywords:** *Schizosaccharomyces pombe*, *Fusarium verticillioides*, Mycotoxin, Fumonisins, Biological control, Yeast

## Abstract

**Abstract:**

*Fusarium verticillioides* poses a high food safety risk worldwide due to its mycotoxin production. Successful control of Fusaria may rely on promising biocontrol agents, including yeasts. Although the fission yeast *Schizosaccharomyces pombe* tolerated *Fusarium* mycotoxins well, including zearalenone, T2, deoxynivalenol, and fumonisins (FUMs), it did not significantly inhibit the growth of *F. verticillioides.* Meanwhile fumonisin B1 (FB1) supplementation did not decrease *S. pombe* cell density in submerged liquid cultures, the colony-forming capability of the yeast was reduced. RNA sequencing showed that *S. pombe* genes involved in cell adhesion and flocculation were downregulated after FB1 exposure. In addition, the expression of several hydrolase genes was also altered. In co-cultures with *F. verticillioides*, genes encoding oxidoreductases and hydrolases and those linked to purine nucleotide metabolisms were downregulated, while the expression of genes involved in membrane and transport processes was increased. The expression of several *F. verticillioides* genes also changed after co-cultivation. Oxidoreductase, transmembrane transport, and purine metabolism genes were upregulated under co-culturing; meanwhile, hydrolase genes, together with carbon metabolism and polysaccharide catabolism genes, were downregulated. Co-cultivation also decreased fumonisin production via the downregulation of genes *FUM19*, *FUM21*, and *FvATFA* encoding the fumonisin transporter, a local Zn(II)2Cys6-type transcriptional regulator and an important global regulator bZIP-type transcription factor, respectively. Although further experiments should clarify the mechanism of the fission yeast-elicited inhibition of fumonisin production, these results may pave the way for the development and implementation of novel, innovative approaches to control mycotoxin production by *F. verticillioides* in the feed and food chain.

**Key points:**

• *0.5 ppm FB1 reduced the colony-forming ability of S. pombe and caused transcriptional changes.*

• *Expression of transport and hydrolase genes changed in yeast during co-cultivation with mold.*

• *Two FUM cluster genes and FvATFA were downregulated in Fusarium co-cultured with S. pombe.*

**Supplementary Information:**

The online version contains supplementary material available at 10.1007/s00253-025-13601-3.

## Introduction

Plant-pathogenic *Fusarium* species often cause severe damage to various crops such as cereals, tomatoes, and bananas (Arie 2019; Jamal et al. 2021; Bahadur 2022). Thereby, these fungi are responsible for great economic losses all over the world. The problem is further exacerbated by the facts that Fusaria belong to a complex, large, and cosmopolitan genus (Arie 2019), whose members are distributed worldwide (Ferrigo et al. 2016; Battilani et al. 2020; Polak-Sliwinska and Paszczyk 2021), and that these fungi can produce mycotoxins depending on various culture and environmental factors (Matić et al. 2013; Ferrigo et al. 2016; Battilani et al. 2020). As these mycotoxigenic molds and their mycotoxins can even survive various feed and food processing procedures, these harmful secondary metabolites can enter the feed and food chain (Glenn 2007; Miedaner et al. 2017), which can cause serious health problems (e.g., hepatotoxic, nephrotoxic effects) both in household animals and humans (Gelderblom et al. 1996; Adam et al. 2017; Ahundov et al. 2019; Ekwomadu et al. 2021).

The harmful mycotoxins fumonisins (FUMs) are produced by several fungi such as *Fusarium verticillioides* and *Fusarium proliferatum*, which often contaminate grains, maize, rice, or grapes (Battilani et al. 2020; Cendoya et al. 2018; Anumudu et al. 2024). *Fusarium verticillioides* produces predominantly four B-series FUMs, designated FB1, FB2, FB3, and FB4 (Cendoya et al. 2018; Battilani et al. 2020; Sultana and Suga 2021). Since fumonisins are structurally similar to the sphingolipid sphinganine (sphingolipids are critical in cell membranes and cell signaling pathways), they can disrupt sphingolipid metabolism by inhibiting ceramide synthase activity (Soriano et al. 2005; Qu et al. 2022). Therefore, it is not surprising that FUM mycotoxin can have a strong physiological effect, increasing the incidence of kidney adenomas and carcinomas in rats, and triggering carcinogenesis in epithelial cells (Voss et al. 2002; Yu et al. 2021). This is probably related to the fact that FUMs promote both cell proliferation and migration, increase the expression of the DNA damage markers, and inhibit the tumor suppressor genes (Soriano et al. 2005; Yu et al. 2021). Because of these harmful effects, the maximum level of FUMs is strictly controlled in several countries (e.g., European Commission Commission Regulation EC No 1881/2006; Ji et al. 2019).

Several strategies have been developed to ensure food safety, ranging from various pre- and post-harvest agricultural practices to chemical control and biological control (Battilani et al. 2020). In the latter case, the growth inhibitory capacity of different microbes is exploited. For example, significant inhibition of *Fusarium* species has been achieved by different yeasts (Papp et al. 2021), such as *Candida krusei* against *Fusarium guttiforme* (Korres et al. 2011), *Pichia anomala* and *Candida parapsilosis* against different Fusaria (Laitila et al. 2007; Fallah et al. 2016), *Kluyveromyces marxianus* against *Fusarium graminearum*, and *F. verticillioides* (Alasmar et al. 2020). Another strategy for food protection can be the elimination of mycotoxins in food and feed by enzymatic degradation with microbial enzymes (Acs-Szabo et al. 2025). This is based on the fact that some microorganisms can produce enzymes such as trichotecene-3-*O*-acetyltransferase, de-epoxidases, carboxylesterases, oxidases, glycosylases, and epimerases that can catalyze the conversion of fungal mycotoxins to less-toxic forms (Kimura et al. 1998; Khatibi et al. 2011; Loi et al. 2017; Azam et al. 2019). Thus, certain microbes can play an important role not only in inhibiting the growth of molds but also in the detoxification and preservation of agricultural products. However, to achieve this often requires strain improvement and genetic modification (Pfliegler et al. 2015).

To successfully combat *Fusarium* spp., a better understanding of the underlying molecular mechanisms of *Fusarium*/*Fusarium* mycotoxins–yeast interactions is needed. Since an in silico bioinformatics search suggested that the genome of the fission yeast* Schizosaccharomyces pombe* contains a large number of potential detoxification genes, including presumably mycotoxin tolerance genes, and this model organism was frequently used in mycotoxin, including patulin, citrinin, and zearalenone (ZEA) cytotoxicity studies before (Papp et al. 2016; Mike et al. 2013; Máté et al. 2014), transcriptomic changes of this yeast triggered by FB1 mycotoxin and its producer *F. verticillioides* were at focus.

## Materials and methods

### Microorganisms and media

*Schizosaccharomyces pombe* Lindner 972 h- {ATCC 843; European Sequencing Consortium (EUPOM)} and *F. verticillioides* (*Gibberella moniliformis* FGSC 7600, genome assembly: GCA_000149555.1 from ENA/EMBL) wild-type strains were used in this study.

YEA (2% glucose, 1% yeast extract, 2% agar; VWR, Radnor, PA, USA) and YEL (YEA without agar) were used as culture media. For the transcriptome analyses, *S. pombe* and *F. verticillioides* cells were cultured in MXGB (Boeira et al. 2000). Antagonism of yeast against *F. verticillioides* was tested on PDB (Potato Dextrose Broth) (Scharlab, Barcelona, Spain), PDA (Potato Dextrose Agar) (Scharlab, Barcelona, Spain), and MXGB media.

### Yeast tolerance to mycotoxins

10^9^ yeast cells from a 16 h pre-culture were inoculated into MXGB medium (final volume 2 ml) prepared with 1 and 2 ppm final concentration of mycotoxins. The BIOPURE deoxynivalenol (DON), T2, zearalenone, and fumonisin B1 (FB1) were purchased from Romer Labs (Tulln, Austria). The Falcon tubes were incubated at 25 ℃ at 100 r.p.m. for 24 h, and the optical density was measured at 570 nm (*n* = 4).

The FB1-treated cells were also spread onto the surface of the MXGB solid medium. The Petri dishes were incubated at 25 ℃, and the colony counts were examined after 6 days. The experiment was repeated four times. Two-sample *T*-test was applied as statistical analysis, and the *P* < 0.05 value was regarded as a significant difference.

### Fumonisin B1 treatment

FUM sensitivity of *S. pombe* was tested with 10^9^ cells and serial dilution of 2 ppm FB1 mycotoxin. The serially diluted FB1-treated cells were spread to MXGB agar plates, and the viable cell numbers were counted at the different mycotoxin concentrations to determine the suitable FUM concentration with sublethal effect on *S. pombe*. Afterward, *S. pombe* cells from an overnight MXGB preculture were inoculated into 30 ml fresh MXGB. The final cell density was set to OD_595nm_ 0.45. The culture was treated with 0.5 ppm FB1 and incubated at 25 ℃ for 1 h. Then, the cells were harvested for transcriptome analysis.

### Preparation of yeast-Fusarium co-cultures

*Fusarium verticillioides* cells were cultured on MXGB solid medium for 5 days at room temperature. 5 × 5 mm hyphal cubes were removed and transferred into 150 ml fresh MXGB medium. After 5 days, the hyphae were transferred into 150 ml MXGB, and 100 µl *S. pombe* cells from an overnight MXGB preculture (3 × 10^7^ cell/ml) were added to the *Fusarium* culture. The co-cultures were incubated further without shaking at 25 ℃ for 2 days.

Since *F. verticillioides* cells floated on the surface of the liquid, while the yeast settled on the bottom of the flask (Supplemental Fig. S1), the yeast and *Fusarium* cells were isolated and centrifuged separately. The wet weight of the samples was measured, and an equal amount of the samples (0.5 g) was used for RNA extraction. The cultures were made in triplicate and three repetitions. Yeast and *F. verticilliodes* monocultures were also analyzed. FUM, pH, and alcohol content of the supernatants were also investigated.

### Analysis of the mycotoxins

The mycotoxin concentrations from culture supernatants were measured with the ELISA method. AgraQuant Fumonisin 0.25/5.0 ELISA kit (Enzyme-Linked Immunosorbent Assay; Romer Labs, Tulln, Austria) was used in a direct competitive assay according to the manufacturer’s instructions, using the Synergy HTX Multimode Reader (BioTek Hungary Ltd., Szigetszentmiklós, Hungary), the samples were measured at 450 nm (*n* = 3; CV% < 5%; coefficient of variation). The analyses were validated by mixing a sterile MXGB medium with known mycotoxin concentrations. FUM LOD (limit of detection) was 0.25 ppm.

Mycotoxin analysis was also performed on PDA, where *F. verticillioides* and *S. pombe* strains were co-cultured. The wet weight of the agar plates was measured, and the agar was homogenized with 5 × volumes of 70% methanol in a Stomacher apparatus (Masticator IUL Instruments)(IUL SA, Barcelona, Spain) for 2 min. Homogenates were filtered through filter paper and then evaporated in a rotary evaporator (VWR LCD Rotary Evaporator) (VWR, Radnor, PA, USA). The residues were taken up in 2 ml aliquots of 70% methanol and centrifuged at 6000 g for 3 min. Aliquots of the supernatants (50 µl of each) were diluted with 950 µl of distilled water according to the manufacturer’s instructions before permitting the ELISA Romer Labs AgraQuant Total Fumonisins (0.25–5 ppm) Quantitative Kit.

### Analysis of volatile organic compounds

For sample preparation, culture supernatants were filtered through folded filter paper (Grade 292, Ahlstrom-Munksjö) (Ahlstrom, Helsinki, Finland), and with Whatman Spartan 13-mm syringe filter (regenerated cellulose, 0.45 μm; GE Healthcare Life Sciences, Little Chalfont, UK) and 1 μl injected to Varian CP 3800GC gas chromatograph loaded with Varian (Varian Inc, Walnut Creek, CA, USA) CP-Wax 52 CB, 30 m × 0.25 mm ID; 0.25 μm Colonna and Flame Ionisation Detector at 230 °C. The carrier gas was 1.0 ml/min helium in constant flow; the injector set was 230 °C with 1:30 split. The schedule: 30 °C (3.0 min); 100 °C/min, 80 °C (hold: 1.0 min); 50 °C/min, 200 °C (9.0 min). The following compounds were tested: acetaldehyde, acetone, methyl acetate, ethyl acetate, methanol, ethanol, and *n*-propanol. LOD was 0.005(v/v)%.

### RNA isolation and RNA sequencing

*Schizosaccharomyces pombe* cells treated with FB1 or grown in co-cultures with *Fusarium*, as well as *F. verticillioides* from co-cultures, and control monocultures were used for RNA sequencing. For RNA extraction, 0.5 g wet weight/sample was used and extracted according to the Lyne method (Lyne et al. 2003). In the case of co-cultures, RNA samples were isolated separately from *F. verticillioides* tissues floating on the surface of the liquid cultures, and yeast cells settled in the bottom of the flasks. However, since *Fusarium* samples may have contained few adherent yeast cells, and settled yeast cells may also have been contaminated by some *Fusarium* hyphal fragments, as well as by macroconidia and microconidia (Ogawa et al. 2019), the RNA samples were pooled (Assefa et al. 2020), i.e., RNAs gained from *Fusarium* and yeast samples separately were mixed in a 1:1 ratio and were sequenced. High-throughput mRNA sequencing was performed on the Illumina platform(Illumina, San Diego, CA, USA), and the quality of the pooled RNA samples was checked on Agilent BioAnalyzer (Santa Clara, CA, USA) using the Eukaryotic Total RNA Nano Kit (Santa Clara, CA, USA), according to the manufacturer’s protocol. Samples with RNA integrity number (RIN) value 7 were used for the library preparation process. Raw reads were aligned to both *S. pombe* and *F. verticillioides* reference genomes (ASM14955v1, ASM294v2). HISAT2 algorithm was applied (Kim et al. 2019). Moderated *T*-test and Benjamini–Hochberg FDR (false discovery rate) statistical analysis were used to identify the genes having significantly altered gene expression (fold change 2 cut-off value).

Library preparations and sequencing runs were performed by UD-GenoMed Ltd and the Genomic Medicine and Bioinformatics Core Facility of the Department of Biochemistry and Molecular Biology, Faculty of Medicine, University of Debrecen, Hungary (Debrecen, Hungary).

### RT-PCR validation

RNA extracted for RNA sequencing was also used for RT-PCR analysis. The isolated RNA was subjected to DNase treatment (M0303; New England Biolabs GmbH, Frankfurt am Main, Germany), and cDNA synthesis was performed with the High-Capacity cDNA Reverse Transcription Kit (Thermo Fisher Scientific—4368814) (Waltham, MA, USA). This cDNA was used for RT-PCR, which was performed with Bio-Rad IQ5 real-time PCR system (Hercules, CA, USA) using SsoAdvanced Universal SYBR® Green Supermix (Bio-Rad, 1725272; Hercules, CA, USA) reagent with the final primer concentrations of 0.1 µM. Serial dilutions of cDNA or gDNA (1/10, 1/100, 1/1000, 1/10000, and 1/10000) were prepared to generate standard curves for each reaction. PCR reactions for the gene expression studies were performed in triplicates. PCR conditions were as follows: 98 °C for 2 min, 40 cycles: 98 °C 5 s, 58 °C 20 s. Melt curves were also generated from 48 to 95 °C. The primers used are presented in Supplemental Table S1.

Data were analyzed with the Bio-Rad iQ5 2.0 software (Hercules, CA, USA) supplied with the qPCR instrument (Hercules, CA, USA). Graphs were generated with GraphPad Prism. *Schizosaccharomyces pombe* gene expression levels were normalized to *sce3* (SPAC17G8.12; exocyst complex subunit Exo1), while *F. verticillioides* gene expression levels were normalized to *tub2* (FVEG_0408; β-tubulin) transcription data, outlying data were removed during analysis. Single culture data were used as the control for double normalization.

### Growth inhibition test

pH of the PDA medium was adjusted to pH 4, pH 5.6, and pH 8. One-day-old yeast cells grown on YEA were streaked onto the surface of PDA opposite to a 6-day culture of *F. verticillioides* mycelium cube (5 × 5 mm). The plates were incubated at 25 ℃, for 6 days. Growth and mycelial development of the *F. verticillioides* were monitored daily for 6 days, and colony size, aerial mycelium, and pigmentation were compared to the mold monoculture.

### Bioinformatics

#### Searching for genes involved in detoxification

The number of genes involved in Detoxification (GO:0098754) was obtained from Amigo database (https://amigo.geneontology.org/) (Carbon et al. 2009).

#### Identification of the gene functions and GO (Gene Ontology) categories

GO categories were obtained from the *S. pombe* database (PomBase) (Harris et al. 2022). The *F. verticillioides* (ASM14955v1) GO data was downloaded from Ensembl Fungi (json file) (https://fungi.ensembl.org/index.html) (Dyer et al. 2025). GO enrichment was performed with the ShinyGO 0.80 (http://bioinformatics.sdstate.edu/go/) (Ge et al. 2020), using default settings (accessed on 27 April 2024).

#### Investigation of *S. pombe*-specific genes

Several *S. pombe*-specific genes showed substantially changed expression levels in the RNA seq. analysis. To establish (or predict) the molecular functions and biological processes in which these *S. pombe*-specific genes are involved, different bioinformatics methods were used. Regular BLASTp analyses were performed at the non-redundant protein database of NCBI (https://blast.ncbi.nlm.nih.gov/Blast.cgi?PAGE=Proteins/) (O’Leary et al. 2024) using the *S. pombe*-specific protein sequences as queries. HMMER searches were done at the website of EMBL-EBI (https://www.ebi.ac.uk/Tools/hmmer/search/phmmer/) using the UniProtKB database; otherwise, default settings were used (Potter et al. 2018). To predict protein function, the DeepGOWeb (https://deepgo.cbrc.kaust.edu.sa/deepgo/) and the LEGO-CSM (https://biosig.lab.uq.edu.au/lego_csm/) tools were used (Kulmanov et al. 2021; Nguyen et al. 2023). Protein interaction analyses were performed in the STRING (https://string-db.org/) database, in the BioGRID (https://thebiogrid.org/) database, and with the PiNT web tool (http://bahlerweb.cs.ucl.ac.uk/PInt/protint_index.htm/) (Pancaldi et al. 2012; Oughtred et al. 2021; Szklarczyk et al. 2023). The first one hundred protein interactors with high RF (random forest) and SVM (support vector machine) scores were taken, and the obtained gene lists were further analyzed by AnGeLi (http://bahlerweb.cs.ucl.ac.uk/cgi-bin/GLA/GLA_input/) and in the PomBase (https://www.pombase.org/query/start_from/gene_list/) focusing on molecular functions and biological processes (Bitton et al. 2015; Rutherford et al. 2024).

### Statistics

Two-sample *T*-test was applied as statistical analysis, and the *P* ≤ 0.05 value was regarded as a significant difference.

## Results

### Detoxification genes in yeasts

**Fig. 1:**
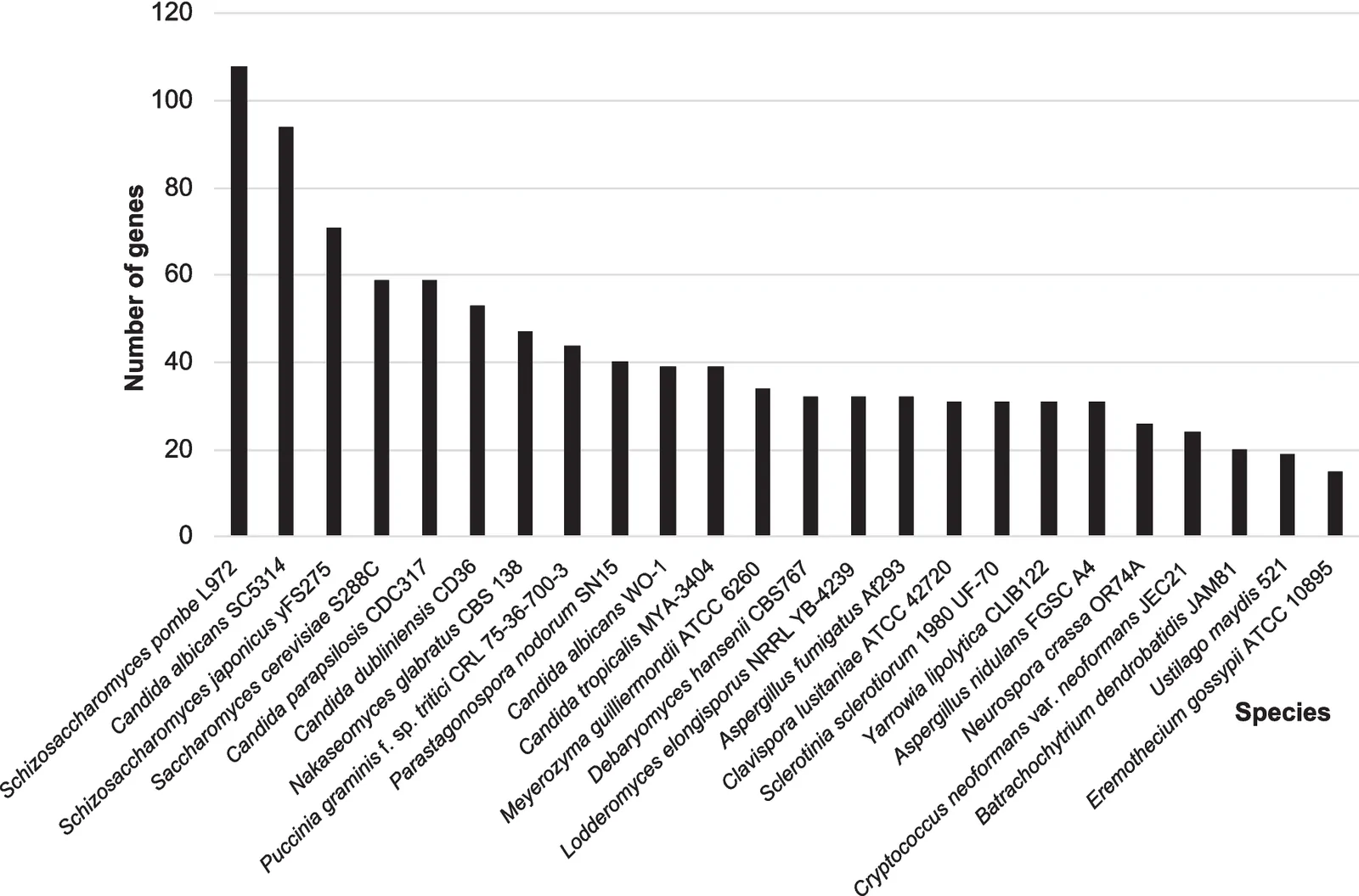
Number of genes involved in detoxification processes in different fungal species (GO:0098754) (Amigo database)

Possible detoxification genes (GO:0098754) involved in processes reducing and/or removing the toxicity of toxic substances were collected from the Amigo database. These genes may encode proteins that keep toxic substances away from their target sites, transport them into compartments, or wrap them into complexes. The results demonstrated that several yeast species harbor detoxification genes, but the number of such genes was highly variable. For example, 15 genes were found in *Eremothecium gossypii*, 94 in *Candida albicans* (SC5314), and 108 in the fission yeast *S. pombe* (Fig. 1).

### *Schizosaccharomyces pombe* tolerates *Fusarium* mycotoxins

Owing to the high number of detoxification genes, *S. pombe* has been selected for further investigation. To evaluate its tolerance to *Fusarium* mycotoxins, fission yeast cells were cultured in the presence of selected *Fusarium* mycotoxins, and the changes in cell density were recorded. According to these observations, *S. pombe* cells tolerated mycotoxins well, and cell density did not decrease in the presence of 1 ppm and 2 ppm DON, T2, ZEA, or FB1 after 24 h (Supplemental Table S2). To further understand the effect of mycotoxins, the colony-forming ability of yeast cells treated with FB1 was also investigated. Although the cell density was not reduced in the previous experiment, viability (colony-forming capability) was reduced by different concentrations of FB1 (statistically significant reduction was 14% for 0.5 ppm FB1 and 49% for 2 ppm FB1) (Supplemental Table S2).

### Transcriptional level response of *S. pombe* cells to fumonisin B1

To understand the changes caused by FB1 in the molecular processes of fission yeast, differentially expressed genes (DEGs) were investigated in the presence of FB1 mycotoxin. RNA sequencing of yeast cells was performed from the culture treated with 0.5 ppm FB1 for 1 h. As shown in Supplemental Fig. S2a and Supplemental Table S3, yeast cells gave a rapid response, and mRNA levels of 166 genes changed significantly compared to the untreated culture. The changes in gene expression were validated in RT-PCR with randomly selected downregulated genes (Supplemental Fig. S2b). The functions and GO categories of DEGs were extracted from the Pombe database (PomBase) (Harris et al. 2022), which showed that the most affected genes were membrane and transport-associated genes, hydrolases, and oxidoreductases (Supplemental Table S3). GO enrichment analysis also highlighted the hydrolases and showed that genes involved in cell adhesion and flocculation were downregulated (Table 1). In addition to the genes mentioned above, many relatively short peptides (65–164 amino acids) coding *S. pombe-*specific genes with unknown functions were also upregulated (Supplemental Table S3). Several different approaches were employed to obtain information on their possible molecular functions. First, the general BLASTp and HMMER searches were performed to find similar sequences. Since none of the sequence searches provided valuable results, prediction of the GO functions using the DeepGOWeb and LEGO-CSM online platforms was performed. Since these approaches did not lead to any results either, or provided us with too many hits with high scores, the possible interaction partners were also searched in the BioGRID, and STRING databases, or using the PiNT web tool. According to these results, the “catalytic activity” of molecular function and “signaling” of the biological process were the most frequently occurring functions for the predicted interacting partners (Supplemental Fig. S3).

**Table 1:** GO enrichment* of the differentially expressed *S. pombe* genes after FB1 treatment

GO biological processes	GO molecular function
Upregulated genes	
Monocarboxylic acid metabolic process (12/106) (GO:0032787)	Hydrolyase activity (6/32) (GO:0016836)
Organic hydroxy compound metabolic process (10/112) (GO:1901615)	Carbon–oxygen lyase activity (6/36) (GO:0016835)
Lactate metabolic process (4/13) (GO:0006089)	Glyoxalase III activity (3/6) (GO:0019172)
Lactate biosynthetic process (3/6) (GO:0019249)	
Downregulated genes	
Flocculation (4/13) (GO:0000128)	Hydrolase activity acting on carbon–nitrogen (but not peptide) bonds (8/63) (GO:0016810)
Adhesion between unicellular organisms (4/13) (GO:0098610)	Hydrolase activity acting on carbon–nitrogen (but not peptide) bonds (6/29) (GO:0016811)
Biological process involved in intraspecies interaction between organisms (4/15) (GO:0051703)	Cell adhesion molecule binding (3/5) (GO:0050839)
Cell–cell adhesion (4/18) (GO:0098609)	Cell adhesion mediator activity (3/5) (GO:0098631)
Cell adhesion (4/24)(GO:0007155)	Glutaminase activity (2/3) (GO:0004359)
Glutamine family amino acid metabolic process (6/58) (GO:0009064)	Asparaginase activity (2/4) (GO:0004067)
Aspartate family amino acid catabolic process (3/11) (GO:0009068)	
Carboxylic acid catabolic process (5/47) (GO:0046395)	
Organic acid catabolic process (5/51) (GO:0016054	
Asparagine catabolic process (2/4) (GO:0006530)	

(Number of genes/pathway genes); *ShinyGO 0.80

### *Schizosaccharomyces pombe*-*F. verticillioides* co-cultures

#### Growth inhibition test and analysis of volatile organic carbons in the supernatant

**Fig. 2:**
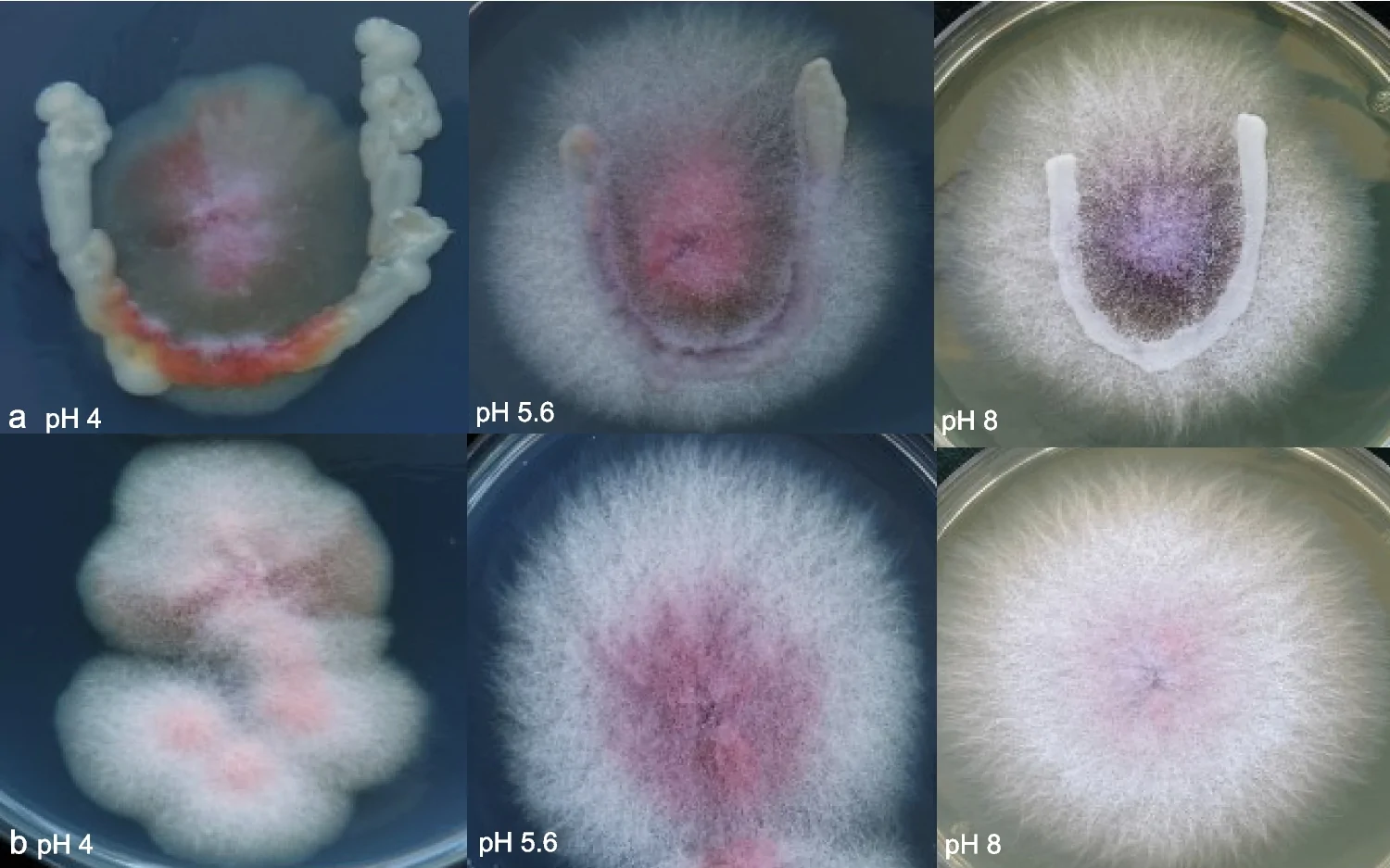
Antagonistic tests. *S. pombe* was unable to inhibit the growth of *F. verticillioides* on PDA medium adjusted to different pH (**a**), as *Fusarium* colonies grew on the yeast and their size was almost similar to the control (**b**). However, *S. pombe* inhibited the aerial mycelia development at pH 4 (**a**). The pigmentation of both species was also changed after co-culturing (**a**) compared to the control (**b**)

To explore the possible biocontrol effect of *S. pombe* and to gain a deeper understanding of the changes caused by *F. verticillioides*, we have prepared co-cultures (Fig. 2). No significant growth inhibition was observed in *F. verticillioides* when the two species were cultured on the same medium (Fig. 2a), as *Fusarium* has grown through the yeast, and its colony size was similar to the control monoculture (Fig. 2b) {average size of colonies: *F. verticillioides*: 5.5 cm (pH 6.5), 6.5 cm (pH 8); *F. verticillioides*-*S. pombe*: 5.15 cm (pH 6.5), 6.0 cm (pH 8)}. However, the development of *F. verticillioides* aerial hyphae was inhibited by *S. pombe* at pH 4 (but not at pH 5.6 and 8) (Fig. 2a). The pigmentation of both species was also changed after co-culturing. The lilac pigmentation of *Fusarium* was darker in the presence of the yeast both at pH 5.6 and 8 (Fig. 2a), compared to the control (Fig. 2b). The yeast also produced pigment in the presence of *F. verticillioides*, but this was pH dependent (red at pH 4, lilac at pH 5.6, and no pigment at pH 8) (Fig. 2a)*.*

Mycotoxin concentration, volatile carbons, and pH were also characterized in the supernatant of the co-cultures. *Fusarium verticillioides* produced 0.488 ± 0.018 ppm FUM, the concentration of which decreased in the presence of *S. pombe* (Table 2). A similar tendency was also found on solid PDA medium (Supplemental Fig. S4); however, these data were not significant.

**Table 2:** FUM content, pH, and ethanol concentration of the *S. pombe*-*F. verticillium* supernatants. The data show the mean ± SD originated from three experiments. Different letters mean significantly different (Student’s *t*-test; *P* < 0.05) results

Cultured species	FUM (ppm)	Ethanol (v/v)%	pH
*Fusarium verticillioides*	0.488 ± 0.018^a^	0.163 ± 0.031^a^	5.31 ± 0.24^a^
*Schizosaccharomyces pombe*	< LOD^b^	1.58 ± 0.14^b^	3.29 ± 0.17^b^
*Schizosaccharomyces pombe*/*Fusarium verticillioides*	< LOD^b^	1.39 ± 0.32^b^	3.71 ± 0.30^b^

Average of three experiments; FUM LOD = 0.25 ppm; ethanol LOD = 0.005(v/v)%

Surprisingly, a low concentration of ethanol (0.163 ± 0.031 v/v%) was also detected from *F. verticillioides* (Table 2); meanwhile, acetaldehyde, acetone, methyl acetate, ethyl acetate, methanol, *n*-butanol, and *n*-propanol were not detectable. In the liquid static co-culture, ethanol production (*P* = 0.0023) and pH (*P* = 0.00079) were significantly different from those found in the liquid monoculture of *F. verticillioides*. Moreover, the pH of the co-culture was also significantly different from that of the *S. pombe* culture (*P* = 0.0311).

#### Transcriptome analysis

To reveal changes in overall gene expression patterns caused by co-culturing, RNA was extracted from both *S. pombe* and *F. verticillioides* cells that were grown in the same culture medium (Supplemental Fig. S1a). RNA sequencing revealed that mRNA levels of 353 DEGs altered significantly in *S. pombe* (Supplemental Table S4; Supplemental Fig. S5a), and 195 upregulated and 158 downregulated DEGs were found in fission yeast cells*.* Gene functions and GO categories were determined, which showed that the expression of genes involved in membrane and transport processes changed significantly, as well as genes with oxidoreductase and hydrolase activities (Supplemental Table S4). GO enrichment analysis also revealed that the DEGs of transport processes were usually upregulated, while hydrolases were downregulated in the presence of *F. verticillioides* (Table 3).

**Table 3:** GO enrichment* of the differentially expressed *S. pombe* genes after co-culturing

GO biological processes	GO molecular function	
Upregulated genes		
Organic acid transport (12/69) (GO:0015849)		Organic acid transmembrane transporter activity (10/55) (GO:0005342)
Organic acid transmembrane transport (11/64) (GO:1903825)		Carboxylic acid transmembrane transporter activity (10/55) (GO:0046943)
Carboxylic acid transmembrane transport (11/64) (GO:1905039)		Transmembrane transporter activity (25/313) (GO:0022857)
Transmembrane transport (28/356) (GO:0055085)		Transporter activity (26/349) (GO:0005215)
Negative reg. of protein kinase activity (4/9) (GO:0006469)		Organic anion transmembrane transporter activity (9/62) (GO:0008514)
		Pentosyltransferase activity (5/20) (GO:0016763)
		Demethylase activity (4/12) (GO:0032451)
Downregulated genes		
Purine nucleobase catabolic process (5/6) (GO:0006145)		Oxidoreductase activity acting on CH-OH group of donors (13/74) (GO:0016614)
Nucleobase catabolic process (5/6) (GO:0046113)		Oxidoreductase activity acting on the CH-OH group of donors NAD or NA (13/70) (GO:0016616)
Purine-containing compound catabolic process (7/16) (GO:0072523)		*D*-threo-aldose 1-dehydrogenase activity (5/14) (GO:0047834)
Nucleobase metabolic process (8/35) (GO:0009112)		Hydrolase activity acting on carbon–nitrogen (but not peptide) bonds (9/63) (GO:0016810)
Small molecule catabolic process (13/108) (GO:0044282)		Aldo–keto reductase (NADP) activity (4/12) (GO:0004033)
Purine-containing compound metabolic process (14/127) (GO:0072521)		Hydrolase activity acting on carbon–nitrogen (but not peptide) bonds (3/7) (GO:0016810)
Nucleobase-containing small molecule metabolic process (18/202) (GO:0055086)		
Heterocycle catabolic process (16/169) (GO:0046700)		
Cellular nitrogen compound catabolic process (16/171) (GO:0044270)		
Organic cyclic compound catabolic process (16/173) (GO:1901361)		
Aromatic compound catabolic process (14/171) (GO:0019439)		

(Number of genes/pathway genes); *ShinyGO 0.80

After comparing the *S. pombe* transcriptional profile data coming from FB1-treated cultures and co-cultures with *F. verticillioides*, overlapping DEGs could also be identified (Supplemental Table S5). Namely, 38 common DEGs were found in the two treatments, and 28 of these DEGs behaved similarly. Presumably, due to the different conditions (e.g., exposure time), the expression of the other 10 overlapping genes changed in the opposite direction (Supplemental Table S5).

**Fig. 3:**
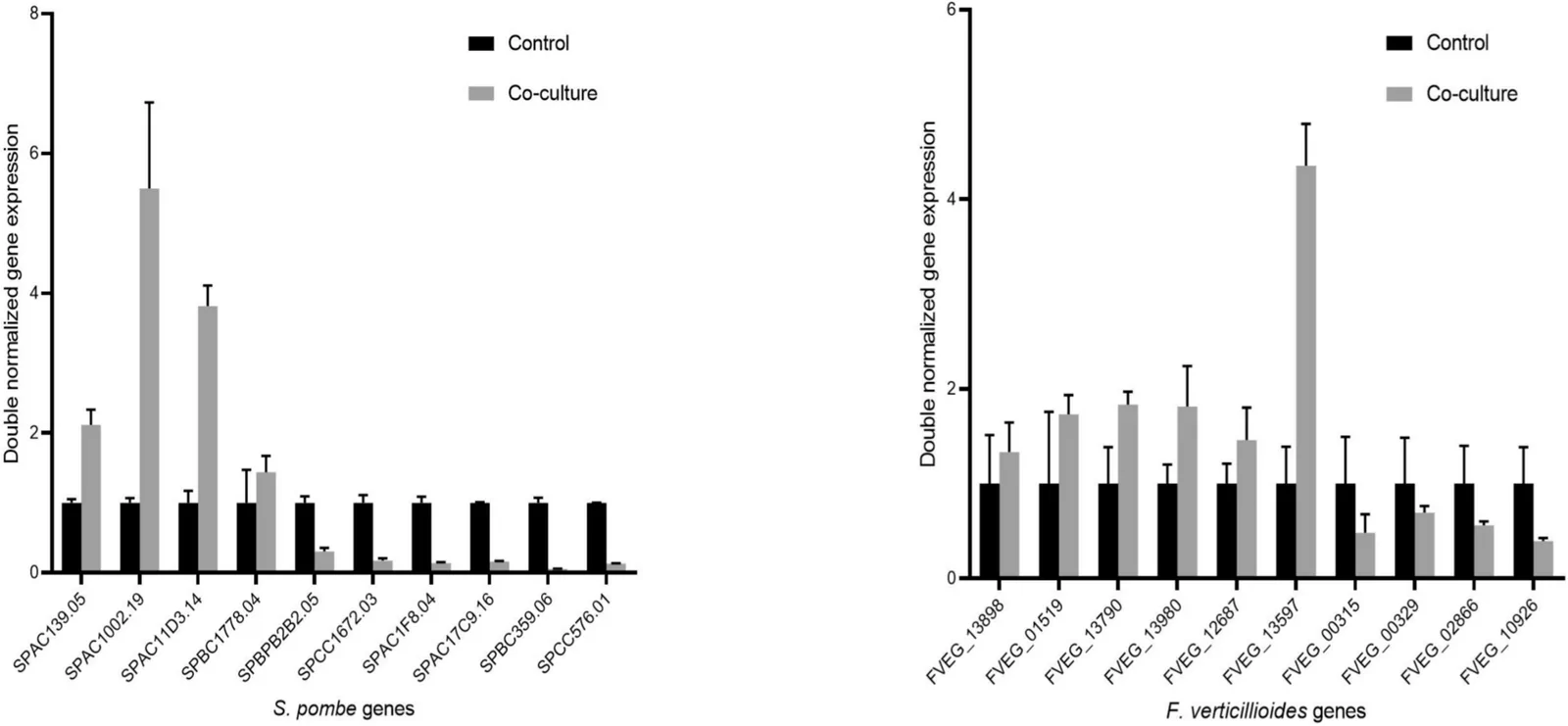
RT-PCR validation of DEGs obtained from co-culture. Gene expression of selected *S. pombe* and *F. verticillioides* genes confirmed the results of RNA sequencing. (FUM cluster genes: FVEG_00329: *FUM19*, FVEG_00315: *FUM21*, FVEG_02866: *FvATFA*) (Control: monoculture, Co-culture: *S. pombe* and *F. verticillioides* cells were cultured in the same medium)

In *F. verticillioides*, the transcription levels of 1870 genes changed significantly under co-culturing, and 880 upregulated and 990 downregulated DEGs were identified (Supplemental Fig. S5b). However, identification of their functions showed that the majority of them are conserved hypothetical proteins with unknown functions (Supplemental Table S6). Nevertheless, the function of 436 genes could be identified (Supplemental Table S6), and the expressions of oxidoreductase genes, transmembrane transport, and purine metabolism genes were upregulated, whereas several hydrolase genes, the carbon metabolism, and polysaccharide catabolism genes were downregulated (Table 4 and Supplemental Table S6). Further analysis of the *F. verticillioides* transcriptom also highlighted that some DEGs were involved in fumonisin biosynthesis. Indeed, *FUM19* (fumonisin ABC transporter) (FVEG_00329), *FUM21* (Zn(II)2Cys6-type local transcription factor) (FVEG_00315) (Alexander et al. 2009; Sun et al. 2019; Sultana and Suga 2021), and *FvATFA* (encoding an *S. pombe* Atf1 and *Aspergillus nidulans* AtfA orthologous bZIP-type global transcriptional regulator) (FVEG_02866) (Szabó et al. 2020) also appeared among the downregulated DEGs (Supplemental Table S6). The transcriptomic changes were validated by RT-PCR (Fig. 3), which confirmed the RNA sequencing results obtained for *S. pombe* and *F. verticillioides* co-cultured cells and also validated the downregulation of genes involved in FUM production.

**Table 4:** GO enrichment* of the differentially expressed *F. verticillioides* genes after co-culturing

GO biological processes	GO molecular function	
Upregulated genes		
Small molecule metabolic process (75/569) (GO:0044282)		Oxidoreductase activity (165/1248) (GO:001649)
Organic acid metabolic process (50/329) (GO:0006082)		Oxidoreductase activity acting on the CH-NH2 group of donors (8/25) (GO:0016638)
Carboxylic acid metabolic process (48/319) (GO:0019752)		Oxidoreductase activity acting on the CH-CH group of donors (17/53) (GO:0016627)
Oxoacid metabolic process (48/322) (GO:0043436)		Oxidoreductase activity acting on single donors with incorporation of molecular oxygen (4/4) (GO:0016701)
Transmembrane transport (89/877) (GO:0055085)		Acyltransferase activity (28/178) (GO:0016746)
ADP metabolic process (6/16) (GO:0046031)		Acyltransferase activity transferring groups other than amino-acyl gr (22/142) (GO:0016747)
Monocarboxylic acid metabolic process (17/83) (GO:0032787)		Transmembrane transporter activity (84/861) (GO:0022857)
Generation of precursor metabolites and energy (16/81) (GO:0006091)		Catalytic activity (366/4021) (GO:0003824)
Glycolytic process (6/14) (GO:0006096)		Ion binding (173/2170) (GO:0005506)
Nucleotide phosphorylation (6/14) (GO:0046939)		Acyl-CoA dehydrogenase activity (3/3) (GO:0003995)
Nucleoside diphosphate phosphorylation (6/14) (GO:0006165)		Vitamin binding (18/118) (GO:0019842)
ATP generation from poly-ADP-*D*-ribose (6/14) (GO:1990966)		Flavin adenine dinucleotide binding (30/218) (GO:0050660)
Serine family amino acid metabolic process (8/30) (GO:0009069)		Carbon–carbon lyase activity (11/59) (GO:0016830)
Purine nucleoside diphosphate metabolic process (6/17) (GO:0009135)		Hydrolyase activity (9/40) (GO:0016787)
Purine ribonucleoside diphosphate metabolic process (6/17) (GO:0009179)		Nitronate monooxygenase activity (4/4) (GO:0018580)
Organic acid catabolic process (12/60) (GO:0016054)		Monooxygenase activity (23/183) (GO:0004497)
Carboxylic acid catabolic process (12/60) (GO:0046395)		
Small molecule catabolic process (17/81) (GO:0044282)		
Fatty acid catabolic process (4/6) (GO:0009062)		
Downregulated genes		
Carbohydrate metabolic process (21/388) (GO:0005975)		Catalytic activity (91/4021) (GO:0003824)
Biological process (89/4847) (GO:0008150)		Hydrolase activity hydrolyzing *O*-glycosyl compounds (18/234) (GO:0004553)
Transmembrane transport (27/877) (GO:0055085)		Hydrolase activity acting on glycosyl bonds (18/245) (GO:0016798)
Transport (31/1189) (GO:0006810)		Transporter activity (29/879) (GO:0005215)
Metabolic process (57/3311) (GO:0008152)		Transmembrane transporter activity (27/861) (GO:0022857)
Establishment of localization (31/1199) (GO:0051234)		Xylanase activity (2/7) (GO:0097599)
Phospholipid metabolic process (8/80) (GO:0006644)		Oxidoreductase activity (30/1248) (GO:0016491)
Phosphorus metabolic process (17/508) (GO:0006793)		Beta-*N*-acetylhexosaminidase activity (2/3) (GO:0004563)
Lipid metabolic process (9/173) (GO:0006629)		*O*-acyltransferase activity (3/12) (GO:0008374)
Phosphate-containing compound metabolic process (16/498) (GO:0006796)		Hexosaminidase activity (2/3) (GO:0015929)
Organophosphate metabolic process (10/224) (GO:0019637)		Phospholipase activity (3/14) (GO:0004620)
Phospholipid biosynthetic process (5/62) (GO:0008654)		Lipase activity (3/26) (GO:0016298)
Polysaccharide catabolic process (5/67) (GO:0000272)		Endo-1,4-beta-xylanase activity (2/6) (GO:0031176)

(Number of genes/pathway genes); *ShinyGO 0.80

## Discussion

Yeast species have a plethora of genes putatively encoding enzymes for mycotoxin degradation, but the species-specific number of possible detoxifying enzyme genes is quite variable. Although aminotransferases and carboxylesterases with hypothetical roles in mycotoxin bioconversion (Heinl et al. 2010; Li et al. 2022) were found in the Pombe Database (Harris et al. 2022), the presence of as many as 108 detoxification genes in the fission yeast *S. pombe* was unexpected (Fig. 1). Because these genes may be involved in the removal of a wide spectrum of toxins, it was hypothesized that *S. pombe* may also have a high tolerance to *Fusarium* mycotoxins. Furthermore, the cytotoxic effects of some well-characterized *Penicillium* and *Fusarium* mycotoxins have been tested in this model organism (Horváth et al. 2012; Mike et al. 2013; Máté et al. 2014; Papp et al. 2016). Therefore, this fission yeast species was selected for further analysis.

The hypothesized mycotoxin tolerance of *S. pombe* was confirmed by a series of growth inhibition assays, which showed no reduction in cell number in the presence of 1 ppm and 2 ppm ZEA, T2, DON, and FB1 (Supplemental Table S2). These data correlated well with the previous results of Boeira et al. (2000). Since high concentrations of mycotoxins may cause loss of viability, such as ZEA in *S. pombe* cells (Mike et al. 2013), or FUM in swine umbilical vein epithelial cells or in porcine endothelial cells (Li et al. 2021; Wang et al. 2022), we also tested cell viability after FUM exposures. Although *S. pombe* cell density did not decrease in the presence of 1 and 2 ppm mycotoxins after 24 h, even 0.5 ppm FB1 significantly reduced the colony-forming ability of *S. pombe* cells (Supplemental Table S2). In addition, genome-wide transcriptional analysis of FB1*-*treated *S. pombe* cells revealed that 1 h 0.5 ppm FB1 mycotoxin exposure affected gene expression, as mRNA levels of 166 genes were significantly altered (Supplemental Table S3 and Fig. S2). This indicates the strong effect of fumonisin and that yeast cells respond even to relatively low concentrations of mycotoxin within a short incubation period. Interestingly, genes involved in cell adhesion and flocculation were mostly downregulated (Table 1). Flocculation of yeasts involves the formation of cell aggregates and special cell–cell interactions depending on environmental factors (Verstrepen and Klis 2006; Kwon et al. 2012) and may be targeted by fumonisins. These data shed light on a possible new aspect of yeast cell wall–mycotoxin interactions because only the mycotoxin adsorption capacity of yeast cell wall was known and exploited so far (Sabater-Vilar et al. 2007; Joannis-Cassan et al. 2011; Qu et al. 2022).

Genes encoding hydrolases that play a key role in the degradation of mycotoxins also showed changed mRNA levels (Supplemental Table S3; Table 1) (Stander et al. 2000; Lyagin and Efremenko 2019; Shukla et al. 2022; Sun et al. 2023). Therefore, we can assume that both yeast cell surface components and degradative enzymes are responsive to FB1 exposures. Several *S. pombe*-specific genes were also upregulated (Supplemental Table S3). As their role is unknown, but in silico predictions suggested that their predicted interacting partners might have a function in catalytic activity and signaling (Supplemental Fig. S3), it would be worth investigating them further.

The growth and transcriptomic changes of the co-cultures provided valuable new pieces of information on the still poorly understood yeast-*Fusarium* interactions. Co-culture on solid PDA medium showed that although *S. pombe* was unable to significantly inhibit the growth of *F. verticillioides*, it was able to inhibit the development of aerial hyphae at pH 4 (Fig. 2a). When the transcriptome of *S. pombe* co-cultured with *F. verticillioides* was investigated, which assumed a longer exposure time to *F. verticillioides* mycotoxins on the yeast side, including fumonisins, a higher number of DEGs (*n* = 353; Supplemental Table S4 and Fig. S5a) were recorded than in yeast monocultures treated with FB1 for a short period of time (*n* = 166; Supplemental Table S3). Interestingly, mRNA levels of hydrolase genes were again changed, mostly downregulated, in co-cultured yeast cells, similar to those observed after FB1 treatment, despite the significant differences in culture conditions (FB1-exposed yeast cells vs. co-cultured cells). The presence of hydrolases among DEGs is consistent with the results obtained in *Aureobasidium pullulans* (Rueda-Mejia et al. 2021), where several secreted hydrolases were found during co-cultivation with *Fusarium oxysporum*, even though the cultivation conditions and species pairs were different from ours. Similarly, genes involved in nucleotide metabolism and translation, or oxidoreductases with altered expression, appeared in both this and a previous experiment (Rueda-Mejia et al. 2021). In addition, genes coding for oxidoreductases, which can act as biocatalytic tools in mycotoxin degradation (Bilal et al. 2022), were downregulated (Table 3). This data supports our earlier assumption that the expression of degradative enzyme genes is affected by FB1. In contrast, transport genes were upregulated (Table 3), which is consistent with Kosawang’s previous report (Kosawang et al. 2014), which also found several transporter genes in the DEG profile of *Clonostachys rosea* after DON and ZEA treatment. Therefore, we can assume that such transporters may be important elements of protection against mycotoxins in yeasts, especially in the case of prolonged exposure.

The comparison of DEGs obtained from FB1-treated yeast cultures and co-cultivations with *F. verticillioides* also revealed that genes characterized by similar GO categories or even identical genes behaved similarly in the two sets of experiments, underlining their responsiveness to fumonisin (Supplemental Table S5).

Co-cultivation affected the yeast and *F. verticillioides* genomes differently, and a higher number of DEGs was detected in *F. verticillioides* than in yeast (Supplemental Table S6 and Fig. S5b). However, many *F. verticillioides* DEGs encoded conserved hypothetical proteins whose role is still unknown, and further experimental analyses are needed to elucidate their function. Considering the *F. verticillioides* DEGs with known or putative functions, they were characterized by GO categories (Supplemental Table S6) partially similar to those of yeast DEGs. This means that many transporters and oxidoreductases were among DEGs on both the yeast and *F. verticillioides* sides; however, *F. verticillioides* oxidoreductase genes were typically upregulated (Table 4). Therefore, we can speculate that oxidoreductases may be involved in the self-defence of *Fusarium* spp. against its own mycotoxins—a hypothesis that needs further studies and verification in the future. So far, only certain *FUM* genes have been confirmed to be involved in self-protection (Krska et al. 2024; Gherlone et al. 2025).

Our additional data showed that co-culture supernatants contained significantly lower fumonisin concentrations than *F. verticiloioides* monocultures (Table 2). A decrease in fumonisin levels was also observed on solid media (PDA); however, this was not statistically significant (Supplementary Fig. S4). This suggests that the composition of culture medium may influence mycotoxin degradation, and therefore, optimization is needed for field/silage studies. These results are consistent with previous findings that the presence of competing fungi had an inhibitory effect on mycotoxin production (Chen et al. 2021; Satterlee et al. 2025) and prompted us to examine whether genes from the well-characterized FUM biosynthetic cluster (Alexander et al. 2009; Sun et al. 2019; Szabó et al. 2020; Sultana and Suga 2021) were present in the differentially expressed genes. Two genes belonging to the FUM cluster, namely *FUM19* (FVEG_00329; fumonisin ABC transporter) and *FUM21* (FVEG_00315; Zn(II)2Cys6-type local transcription factor), as well as *FvATFA* (FVEG_02866; encoding a bZIP-type global transcriptional factor positively regulating FB1 and FB2 productions (Szabó et al. 2020; Leiter et al. 2021)), were downregulated in co-cultured *F. verticillioides* cells, which changes were also validated by RT-PCR (Fig. 3). Similarly, the FUM-related pathway also appeared to be downregulated in the presence of the biological control agent *Streptomyces* sp. AV05 (Strub et al. 2021).

As regards the role of the transcriptional factor genes in fumonisin production, previous results have shown that deletion of the *FUM21* and *FvATFA* genes in *F. verticillioides* led to impaired fumonisin production (Sun et al. 2019; Janevska et al. 2020; Szabó et al. 2020). Thus, the decrease in their gene expression may well explain the observed decrease in fumonisin production (Table 2). Since these regulatory genes also affect the function of other genes, for example, *FUM21* affects the genes *FUM17* and *FUM18* (encoding ceramide synthases; Janevska et al. 2020), and the deletion of the *FvATFA* caused pleiotropic changes, including downregulation of *FUM1* (the polyketide synthase gene) and *FUM8* (encoding α-oxoamine synthase; Szabó et al. 2020), these genes could be good drug targets if we want to reduce fumonisin production.

However, the decrease in fumonisin levels contradicts the previous finding that deletion of the ABC transporter *FUM19* gene caused an increase in fumonisin B1 levels (Proctor et al. 2003; Janevska et al. 2020). In this sense, the downregulation of *FUM19* would have increased fumonisin levels, but this did not occur. One possible explanation could be that, since this gene is a transporter gene, it regulates not only intracellular and secreted fumonisin (Janevska et al. 2020) but also the transport of other compounds that can influence fumonisin synthesis. This idea is indirectly supported by the observations that, for example, sugar and amino acids influenced the production of fumonisin B1 in *F. fujikuroi* (Jimenez 2003), and that deletion of the *FUM19* gene in *F. proliferatum* affected other processes, as well, e.g., increased conidia production (Sun et al. 2019). In other words, this transporter gene may indirectly regulate the FUM cluster; however, this hypothesis requires further evidence.

It is not clear at the moment how co-culturing with fission yeast downregulated the FUM biosynthetic cluster genes and *FvATFA* in *F. verticillioides*. For example, ethanol produced by the yeast cells may interfere with fumonisin production in *F. verticillioides*, similarly to aflatoxin production in *Aspergillus flavus* (Ren et al. 2020). However, it should be noted that *F. verticillioides* also produced low amounts of ethanol in liquid culture under anaerobic conditions, as did *F. oxysporum* (Xiros and Christakopoulos 2009; De Almeida et al. 2013). In addition, several other, yet-to-be-identified antimycotic and/or anti-mycotoxigenic compounds might also be produced by fission yeast at 48 h co-incubation, as baker’s yeast extract itself influenced *F. fujikuroi* FB1 production (Sørensen and Sondergaard 2014).

Another explanation can be that other factors may contribute to the reduction in fumonisin production capacity of *F. verticillioides*, such as the fact that *S. pombe* reproduces much faster and consumes the nutrients in the culture medium, as the amount of nutrients can influence fumonisin production (Shim and Woloshuk 1999; Jimenez 2003). The limited nutrient supply may also be indicated by the fact that co-cultivation triggered stronger pigment production in *Fusarium* (Fig. 2), which is a process dependent on environmental factors, and, for example, nutrient deficiency triggered bikaverin production in *F. fujikuroi* (Limón et al. 2010).

Summing it up, transcriptional changes in co-cultures of *S. pombe* and *F. verticillioides* were recorded for the first time in both interacting species concomitantly. These data may contribute to the identification of genes and gene products that may be suitable for fumonisin detoxification and/or inhibition of its production. Shedding light on the fission yeast compounds interfering with FUM production at the level of *FUM19*, *FUM21*, and *FvATFA* gene expressions in *F. verticillioides* may also help us develop novel *Fusarium* mycotoxin control technologies to protect the feed and food chain from harmful fumonisins (Kushiro 2008; Battilani et al. 2020; Polak-Śliwińska and Paszczyk 2021).

## Supplementary Information

Below is the link to the electronic supplementary material.

Supplementary file1 (XLSX 436 KB)

Supplementary file2 (PDF 761 KB)

## Data Availability

All data generated or analyzed during this study are included in this published article and its supplementary information files. The transcriptome data sets are also available in the Gene Expression Omnibus database (GEO; [http://www.ncbi.nlm.nih.gov/geo](http:/www.ncbi.nlm.nih.gov/geo)/; accessed on 11 June 2024) with the following accession number: GSE269603.
