# Supplementary material for: Transcriptome changes of fission yeast cells exposed to fumonisin B1 or co-cultured with Fusarium verticillioides
Source: Appl Microbiol Biotechnol. 2025 Oct 1;109(1):211. doi: 10.1007/s00253-025-13601-3 (PMC12488821; doi:10.1007/s00253-025-13601-3)
Supplement: Supplementary file 2 — Supplementary file2 (PDF 761 KB) [file 253_2025_13601_MOESM2_ESM.pdf]

Applied Microbiology and Biotechnology

Supplemental Figures

Transcriptome changes of fission yeast cells exposed to fumonisin B1 or co-cultured with *Fusarium verticillioides*

László Attila Papp[1], Lajos Acs-Szabo[1,2], Szilvia Kovács[3], Cintia Adácsi[3], Gyula Batta[1], Tünde Pusztahelyi[3], István Pócsi[4,5], and Ida Miklós[1]*

[1]Department of Genetics and Applied Microbiology, Faculty of Science and Technology, Institute of Biotechnology, University of Debrecen, Egyetem tér 1, H-4032 Debrecen, Hungary

[2]Department of Botany, Faculty of Science and Technology, Institute of Biology and Ecology, University of Debrecen, Egyetem tér 1, H-4032 Debrecen, Hungary

[3]Central Laboratory of Agricultural and Food Products, Faculty of Agricultural and Food Sciences and Environmental Management, University of Debrecen, Böszörményi Street 138, H-4032 Debrecen, Hungary

[4]Department of Molecular Biotechnology and Microbiology, Faculty of Science and Technology, Institute of Biotechnology, University of Debrecen, Egyetem tér 1, H-4032 Debrecen, Hungary

[5]HUN-REN-UD Fungal Stress Biology Research Group, Egyetem tér 1, H-4032 Debrecen, Hungary

*Correspondence to: Ida Miklós, miklos.ida@science.unideb.hu, ORCID ID 0000-0002-7074-4019

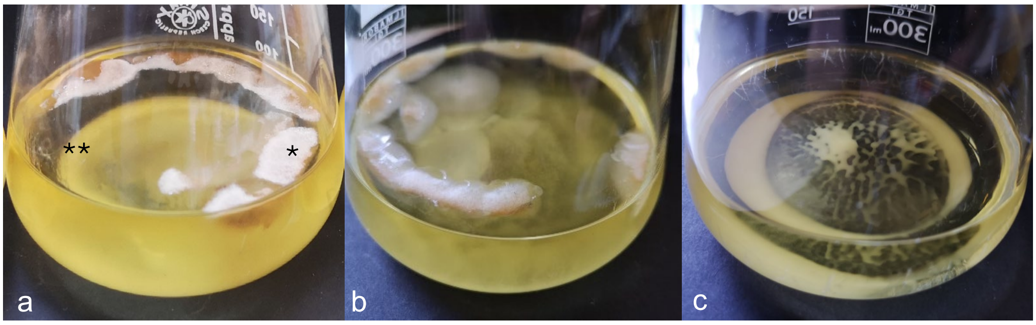

Fig. S1 *S. pombe*–*F. verticillioides* co-cultures were prepared (a). The cultures were incubated without shaking, at 25°C, for 2 days (*: *F. verticillioides* cells floated on the surface of the liquid, **: *S. pombe* cells settled on the bottom of the flask). Controls: *F. verticillioides* (b), and *S. pombe* monocultures (c)

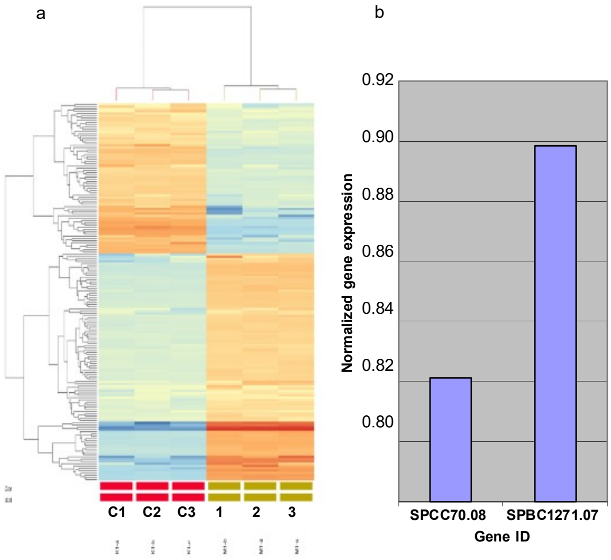

Fig. S2 RNA sequencing of the *S. pombe* cells treated with 0.5 ppm FB1. Heatmap of genes with statistically significantly altered mRNA levels (a); C1, C2, C3: control, 1, 2, 3: FB1-treated samples. Blue color: down-regulated genes, reddish color: up-regulated genes. RT-PCR analysis of randomly selected down-regulated genes confirmed the results of RNA sequencing, as their normalized gene expression was below 1 (b)

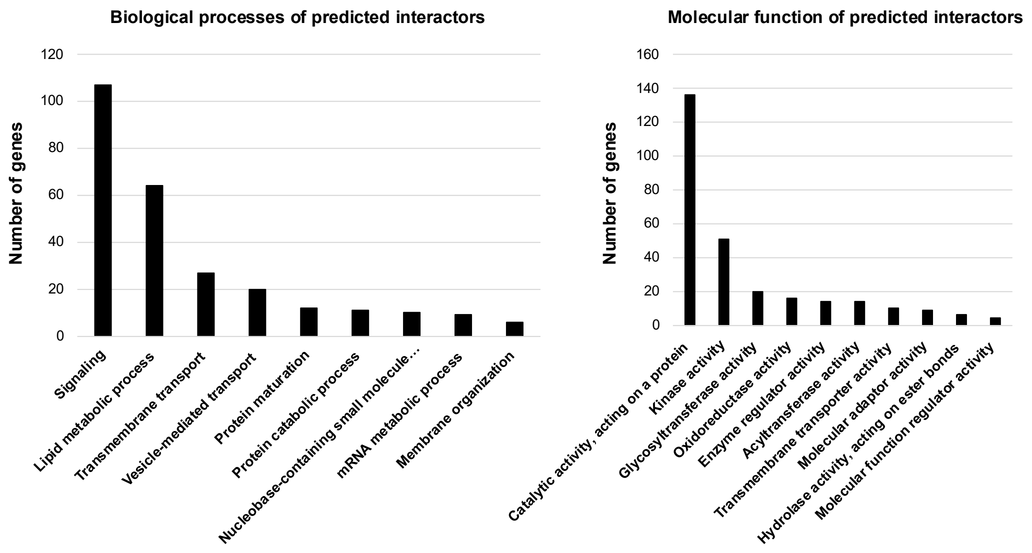

Fig. S3 Biological processes and molecular functions of the predicted interactors of the differentially expressed *S. pombe*-specific genes after FUM treatment. Bioinformatics analysis suggests that most interactor proteins may have a "signal transduction" and "catalytic activity" function

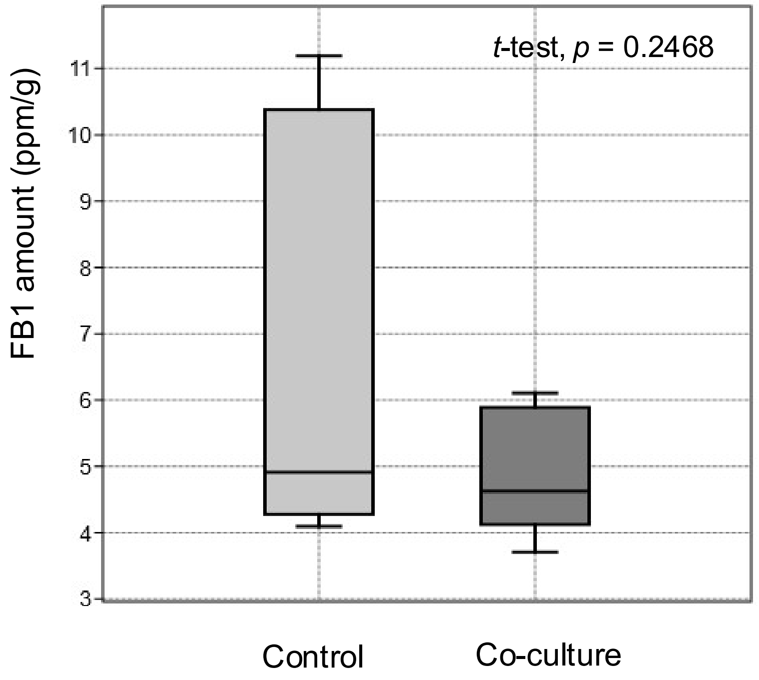

Fig. S4 Fumonisin production of *F. verticillioides* was reduced when co-cultured with *S. pombe* (PDA, pH 4.0, room temperature, 6 days); however, it was not significant. Control: *F. verticillioides* monoculture, Co-culture: *F. verticillioides* and *S. pombe* were grown on the same Petri dishes (n = 5)

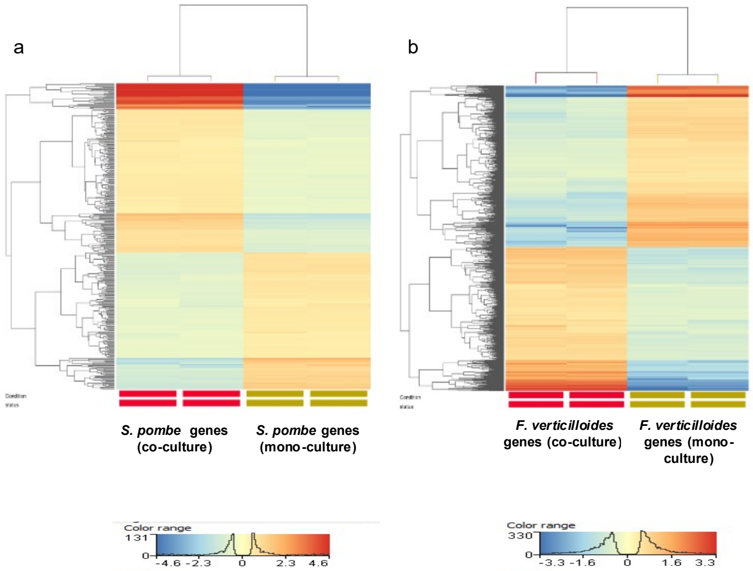

Fig. S5 *S. pombe–F. verticillioides* co-cultures were prepared and transcriptional profile analyses were performed from the yeast and *Fusarium* cells. Heatmap of the significantly expressed *S. pombe* (a) and *F. verticillioides* (b) genes. Blue color: down-regulated genes, reddish color: up-regulated genes
